# Combined single-cell RNA-seq and bulk RNA-seq construction of M2 TAMs signature for predicting HNSCC prognosis and immunotherapy

**DOI:** 10.3389/fimmu.2025.1620931

**Published:** 2025-08-12

**Authors:** Jiale Wang, Huan Li, Mingrui Shi, Chenghao Ren, Wu Wei, Qi Zhao, Xinxin He, Zihui Yang, Jianhua Wei, Xinjie Yang

**Affiliations:** State Key Laboratory of Oral and Maxillofacial Reconstruction and Regeneration, National Clinical Research Center for Oral Diseases, Shaanxi Clinical Research Center for Oral Diseases, Department of Oral and Maxillofacial Surgery, School of Stomatology, The Former Fourth Military Medical University, Xi’an, China

**Keywords:** single-cell RNA sequencing, tumor-associated macrophages, head and neck squamous cell carcinoma, weighted correlation network analysis, immune profile

## Abstract

Tumor associated macrophages (TAMs) in Head and neck squamous cell carcinoma (HNSCC), particularly M2-polarized subtypes, are pivotal drivers of tumorigenesis, angiogenesis, and metastasis, contributing to adverse clinical outcomes. Current prognostic markers lack precision, underscoring the need for novel biomarkers and risk stratification models. Single-cell RNA sequencing (scRNA-seq) was applied to profile the transcriptional landscape of TAMs in HNSCC at single-cell resolution. 1,208 M2 TAMs were integrated from scRNA-seq data with bulk RNA sequencing to identify molecular signatures. Weighted correlation network analysis (WGCNA) and Uniform Manifold Approximation and Projection (UMAP) analysis were applied to dissect TAMs heterogeneity and interactions within the tumor microenvironment. *In vivo* experiments validated the efficacy of the prognostic signature model. In this study, high infiltration of M2 TAMs was strongly associated with advanced clinical stages, lymph node metastasis, and reduced overall survival (P<0.001). TCGA datasets were utilized for cross-platform verification. Multivariate Cox regression and survival analyses were performed to establish prognostic relevance. 11 prognostic signature genes (FCGBP, GIMAP5, WIPF1, RASGEF1B, GIMAP7, IGFLR1, GPR35, NCF1, CLECL1, HEXB, IL10) were identified through integrative analysis, which formed the basis of a robust risk stratification model. The distribution of biomarkers in the high-risk group, as determined by the signature we constructed, can serve as a better indicator for assessing poor prognosis. In clinical samples, prognosis signature has the potential to predict the prognosis effectively in patients with HNSCC.M2 TAMs-driven prognostic signature for HNSCC offers a clinically actionable tool for risk stratification and outcome prediction.

## 1 Introduction

Head and neck squamous cell carcinoma (HNSCC) is the sixth most prevalent cancer globally and is characterized by its aggressive behavior and poor prognosis (1). The treatment strategy for HNSCC is a comprehensive sequential treatment with surgery as the primary approach and adjuvant treatments include adjuvant radiotherapy, chemotherapy and others (2). However, despite recent advancements in therapeutic approaches, such as multidisciplinary approaches and targeted immunotherapy, the 5-year overall survival rate for HNSCC patients remains approximately 50% (3). Furthermore, the limited accuracy of existing prognostic markers for HNSCC hinders the development of more effective diagnosis tools.

Tumor-associated macrophages (TAMs) play a pivotal role in tumorigenesis, angiogenesis, invasion metastasis, all of which contribute to adverse clinical outcomes (4, 5). In colon cancer, distinct TAMs populations inhabit separate microenvironments, predicting divergent prognostic outcomes (6). In gastric cancer, metabolic features of M2 macrophages identified through database analysis, are associated with the poor prognosis (7). While previous research establish that TAMs contribute to the poor prognosis of patients with HNSCC, the underlying mechanisms remains poorly understood (7).

Single-cell RNA sequencing (scRNA-seq) technique offers an advanced methodology to analyze transcription at the single-cell level. This technique allows for a more precise exploration of the molecular signatures involved in tumor development and progression, compared to traditional methods (8). This advantage bolsters the confidence in using single-cell sequencing as a prognostic tool for cancer (9). The scRNA-seq technique has been used to investigate interactions between immune and non-immune cells (10) and has revealed the diversity of TAMs and their role in tumor progression (11).

Several studies have demonstrated the utility of database analysis of TAMs features in predicting cancer prognosis (6, 12–14). In this study, we investigated, for the first time, the prognostic application of TAMs in HNSCC using single-cell sequencing technology. TAMs signature was screened by integrating both bulk and single-cell RNA sequencing to predict prognosis and guide immunotherapy. The results of the present study provide valuable insights into the molecular mechanisms underlying M2 TAMs in HNSCC, elucidate the immune landscape of this malignancy and identify potential therapeutic targets. Our research established a robust prognostic prediction model for HNSCC, contributing to more precise diagnosis and treatment.

## 2 Materials and methods

### 2.1 Patients and samples

The study was approved by the Stomatology Hospital of Air Force Medical University, and all patients participated in this study had signed the informed consent. Twenty patients with HNSCC who underwent surgery between January 2023 and January 2024 provided primary tumor tissues. The diagnosis of HNSCC was based on WHO Classification of Head and Neck Tumors (5th edition) and the TNM staging system (8th edition, UICC). The collected tissues were fixed in 10% neutral-buffered formalin and embedded in paraffin for subsequent pathological examination and staining.

### 2.2 Acquisition and preprocessing of data

Gene expression datasets were obtained from the Gene Expression Omnibus (GEO) repository under accession numbers GSE65858 (bulk RNA-seq), GSE150430 (single-cell RNA-seq), and GSE123813 (single-cell RNA-seq). Fifteen primary tumor samples from GSE150430 were included in this study (**Table 1**). RNA-seq FPKM expression profiles, overall survival (OS) data, and clinical annotations for HNSCC were retrieved from the National Cancer Institute (NCI)’s Genomic Data Commons (GDC).

**Table 1:** Quality control of single-cell transcriptome data and genetic.

Dataset ID	Data type	Samples number	Purpose
TCGA- HNSCC	bulk	494	Screening M2 macrophage-related module genes, model construction, training set
GSE65858	bulk	270	Model validation, validation set
GSE150430	scRNA	15	Screening characteristic genes of TAM cells
GSE123813	scRNA	4	Evaluate immunotherapy

### 2.3 Screening

Fifteen primary samples from GSE150430 were processed using the R software package and underwent quality control in GEO. Two thousand highly variable genes were identified using the “FindVariableFeatures” algorithm. Principal component analysis (PCA) was conducted on these HVGs, with the top 50 principal components retained for subsequent analyses. TAMs associated marker genes were identified using the “FindAllMarkers” function(P<0.05). WGCNA constructed co-expression modules (minimum size=30 genes) through soft thresholding. Finally, module eigengenes were intersected with TAMs signature genes identified through single-cell analysis to pinpoint M2 TAMs related genes.

### 2.4 Survival analysis of the proportion of macrophage infiltration

The relative abundance of M1 and M2 macrophages was quantified using the XCell algorithm. Samples were stratified into high-risk and low-risk groups based on macrophage infiltration levels, applying a median cut-off value derived from the R package XCell. Kaplan-Meier survival analysis with log-rank testing was performed to evaluate the correlation between macrophage infiltration density and overall survival (OS) in HNSCC patients.

### 2.5 The development of the prognostic signature related to M2 TAMs

Univariate Cox regression was performed to identify M2 TAMs-related prognostic genes based on the survival curve (*P<0.05*). The R package was employed to construct a LASSO Cox regression model to identify prognostic factors. A risk score model was developed by weighting key prognostic factors with LASSO regression coefficients to predict survival.


$$Score=\sum_{i=1}^{n}exp_{i}\times coef_{i}$$

Based on corresponding scores, fifteen samples were classified into high-risk group and low risk group and survival curve were visualized using the Kaplan-Meier method with the log-rank test. The receiver operating characteristic (ROC) curve was adapted to evaluate the predictive performance of the scoring system, and the area under the curve (AUC) was visualized with the R package time ROC. Univariate and multivariate Cox regression analyses were performed to evaluate the independent prognostic value of the risk score.

### 2.6 Predicting drug sensitivity

The half-maximal inhibitory concentration (IC50) values for training set samples were estimated using the Phenotype algorithm implemented in the R package Predict (v1.2.3), with drug sensitivity data sourced from the Genomics of Drug Sensitivity in Cancer (GDSC) database (version 2.0; PMID: 22000000).

### 2.7 Gene set variation analysis and functional annotation

Gene Ontology (GO) and Kyoto Encyclopedia of Genes and Genomes (KEGG) pathway enrichment analyses were performed using the R package clusterProfiler (v4.0.1) to functionally annotate the signature genes. According to the enrichment analysis result, differences in immune function between the high-risk group and low-risk group were compared using Gene Set Variation Analysis (GSVA) and Gene Set Enrichment Analysis (GSEA). Immune cell infiltration across subgroups was compared using the Wilcoxon test and the ssGSEA (single-sample gene-set enrichment analysis) algorithm. The relative abundance of 28 immune cell subsets (e.g., activated CD8+ T cells, dendritic cells, and macrophages) within the tumor microenvironment (TME) were quantified.

### 2.8 Immunofluorescence

Tissue sections were fixed with 4% paraformaldehyde in PBS for 20 minutes at room temperature. Next, the membranes were blocked with 1% BSA for 2 hours at room temperature. The membranes were then incubated with primary antibodies against CD163 and iNOS at 4°C overnight. The results were observed using a laser scanning confocal microscope.

### 2.9 Immunohistochemistry

Sections and TMAs were stained with or incubated with primary antibodies using the Elivision™ Plus Polymer HRP immunohistochemistry kit (Maxim, Fujian, China). The following antibodies were used: anti-FCGBP (ab121199, Abcam, 1:500), anti-GPR35 (ab150635, Abcam, 1:300), GIMAP7 polyclonal antibody (Proteintech, 1:500), WIPF1 polyclonal antibody (Proteintech, 1:500), RASGEF1B polyclonal antibody (Proteintech, 1:300), p47 phox polyclonal antibody (Proteintech, 1:400), HEXB polyclonal antibody (Proteintech, 1:400), IL-10 monoclonal antibody (Proteintech, 1:500), CLECL1 monoclonal antibody (Proteintech, 1:500),GIMAP5 polyclonal antibody (AtaGenix,1:800),and CLECL1 polyclonal antibody(AtaGenix,1:800). The score of each section was classified into 0–4 by the ImageJ software based on the intensity and the positive rate of stained cells.

### 2.10 Animal experiments

All experimental protocols were approved by the Institutional Animal Care and Use Committee (IACUC) of the State Key Laboratory, Air Force Military Medical University. Female BALB/c nude mice (6-week-old, n=20) were subcutaneously inoculated with SCC9 (1–5 × 10^7^cells/mouse) into the left forelimb. One-week post-inoculation, mice were randomized into four groups: the experimental group first (n=5) received lipopolysaccharide (LPS) (20 mg/kg, 200 μL), group second (n=5) was administered recombinant IL-4 (20 mg/kg, 200 μL), while the control group third (blank) (n=5) and forth(n=5) with (SCC+Normal Saline,200 μL). LPS and IL-4 treatments were administered to the mice one week after tumor cell injection, when the subcutaneous tumorigenesis model was successfully established with a tumor volume (V) > 100 mm³ (to avoid the impact of early intervention on tumor formation). The experiment was terminated when the tumor volume (V) < 1500 mm³, and all mice were euthanized on day 27 in accordance with the ethical norms for animal experiments. Following confirmation of tumor formation, injections were administered three times at 48-hour intervals. Tumor tissues were harvested for immunohistochemical (IHC) analysis of CD68 (pan-macrophage marker), CD163 (M2 macrophage marker), and iNOS (M1 macrophage marker) expression. Prognostic significance was evaluated based on tumor weight as a key measure of tumor progression in HNSCC.

### 2.11 Statistical analysis

Statistical analyses were conducted using R software (version 4.1.2). The Wilcoxon rank-sum test was applied to compare differences between two groups, while the Kruskal-Wallis’s test was used for comparisons involving multiple groups. Survival curves for prognostic analysis were generated using the Kaplan-Meier method, and the log-rank test was used to determine the significance of differences. In graphical representations, significance levels were denoted as follows: ns (not significant, *P* > 0.05), *(*P* < 0.05), ** (*P* < 0.01), and *** (*P* < 0.001).

## 3 Result

### 3.1 M2 TAMs leads to poor prognosis in patients with HNSCC

**Figure 1:**
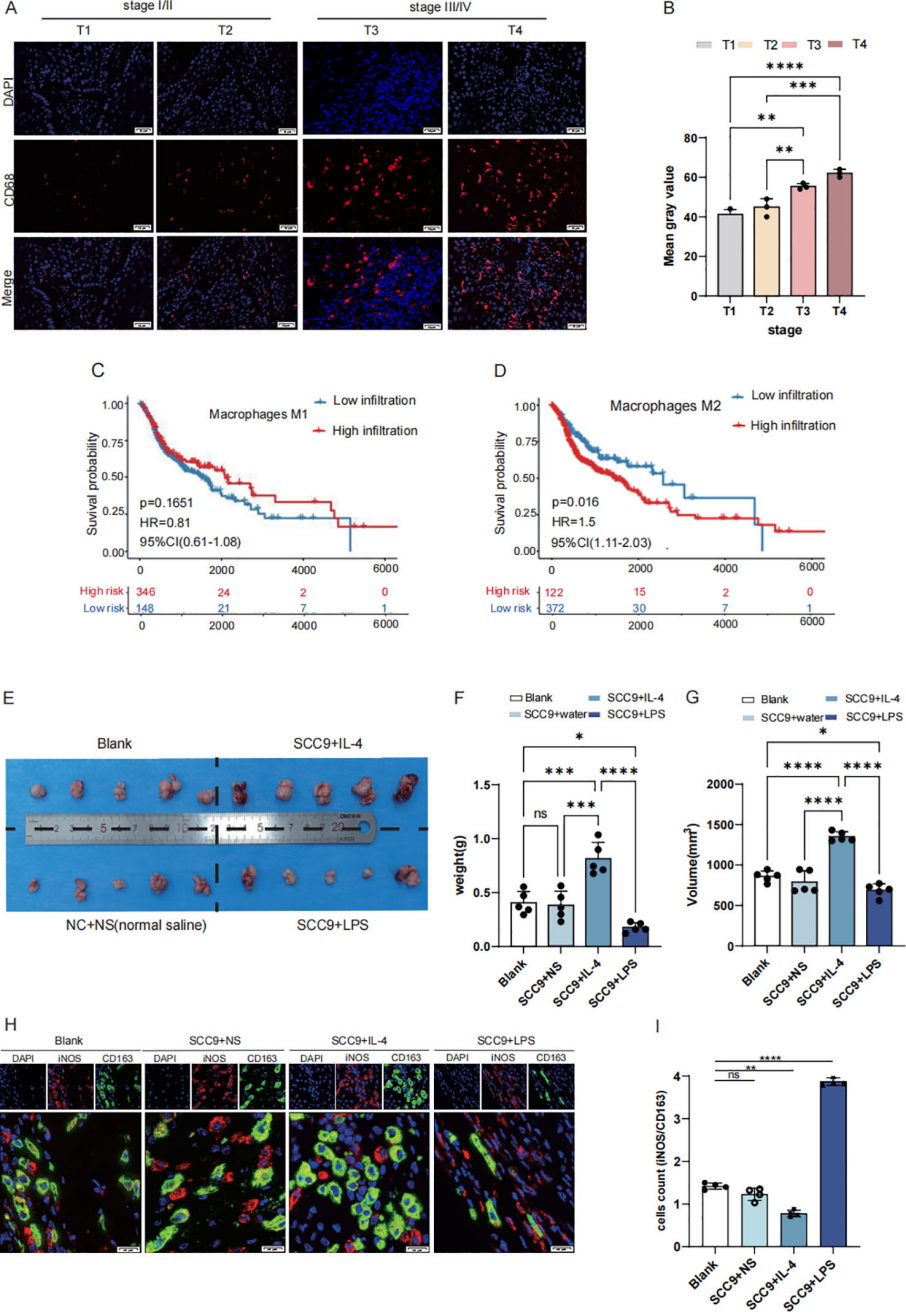
M2 macrophages exhibit high infiltration in HNSCC with poor prognosis. **(A, B)** High expression CD68 in stage III/IV (selected from the clinical patient case database, Scale bar: 50 μm) **(C)** The OS of patients with high expression M1 type macrophage **(D)** The OS of patients with high expression M2 type macrophage **(E)** Establishing a mouse subcutaneous tumor model with the following groups: Blank, SCC9+NS (Normal sailine),SCC9+LPS,SCC9+IL4 **(F)** The weight of BALC/c nude mice cancer samples: SCC + IL4 treatment group exhibited a larger tumor volume compared to the SCC + NS group, while the SCC + LPS treatment group showed a smaller tumor volume **(G)** The volume of BALC/c nude mice cancer samples **(H, I)** Based on the iNOS/CD163 ratio, macrophages in SCC tumors exhibited a greater tendency toward M1 polarization upon LPS treatment, whereas IL-4 treatment promoted more pronounced M2 polarization (Scale bar: 20µm; ns, not significant, P > 0.05), Asterisk (*) indicates statistical significance: *p < 0.05, **p < 0.01, ***p < 0.001.

TAMs infiltration was more abundant in stage III/IV tumors in stage I/II based on the expression of CD68 in immunofluorescence (**Figures 1A, B**). Survival curve based on TCGA data confirmed that M2 macrophages are strongly associated with poor prognosis in malignancies, while M1-type macrophages associated with better prognosis (**Figures 1C, D**). Mouse subcutaneous tumor model was constructed to reveal the infiltration situation (**Figure 1E**). The weight and volume of SCC9 with IL-4 which means M2 TAMs rich infiltration group were higher than SCC9 with LPS which means M1-type TAMs rich infiltration, control and blank (*P*<0.05) (**Figures 1F, G**). Immunofluorescence staining also showed that M2 TAMs infiltration was richer based on the expression of CD163 in SCC9 with IL-4 group than in SCC9 with LPS (*P*<0.05) (**Figures 1H, I**).

### 3.2 Prognostic genes associated with the M2 TAMs

WGCNA was employed to screen M2 macrophages-related genes to explore their association with the prognosis of HNSCC. As shown in **Supplementary File 1**, the WGCNA results identified M2 macrophage-related genes in HNSCC and revealed 25 optimal modules. Among these 25 optimal modules, the blue module, which exhibited the highest Pearson correlation coefficient, was selected for downstream analysis and contained 778 genes.

**Figure 2:**
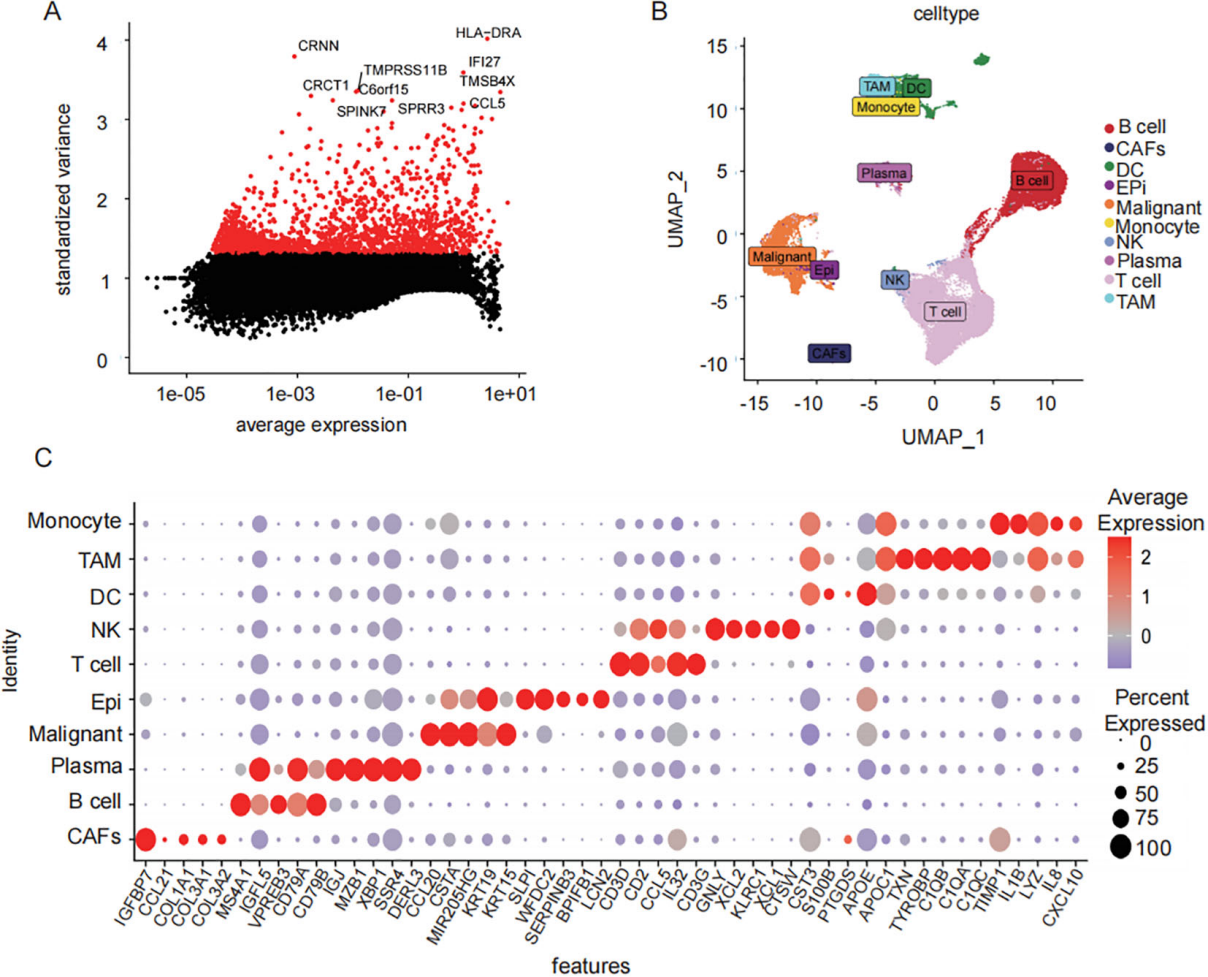
Single cell data TAMs characterization gene screening. **(A)** scatter-plot of highly variable CRNN, CRCT1, and HLA-DRA **(B)** UMAP results demonstrated the spatial distribution patterns of heterogeneous immune cell populations within the tumor microenvironment. **(C)** distribution of sample cells after removal of batch effects different cell types top5 characteristic gene gas (bubble map).

From 46,001 single-cell transcriptomes, the top 2,000 highly variable genes (HVGs), including CRNN, CRCT1, and HLA-DRA, were selected for further analysis (**Figure 2A**). Uniform Manifold Approximation and Projection (UMAP) visualization was used to display the top 50 principal components (PCs) and reveal distinct cellular clusters. The Harmony algorithm was applied to correct batch effects (**Figure 2B**). Cell-type-specific marker genes were identified using the Find All Markers function and the top five markers per cluster were visualized (**Figure 2C**). In total, 1,208 TAMs-specific signature genes were identified.

**Figure 3:**
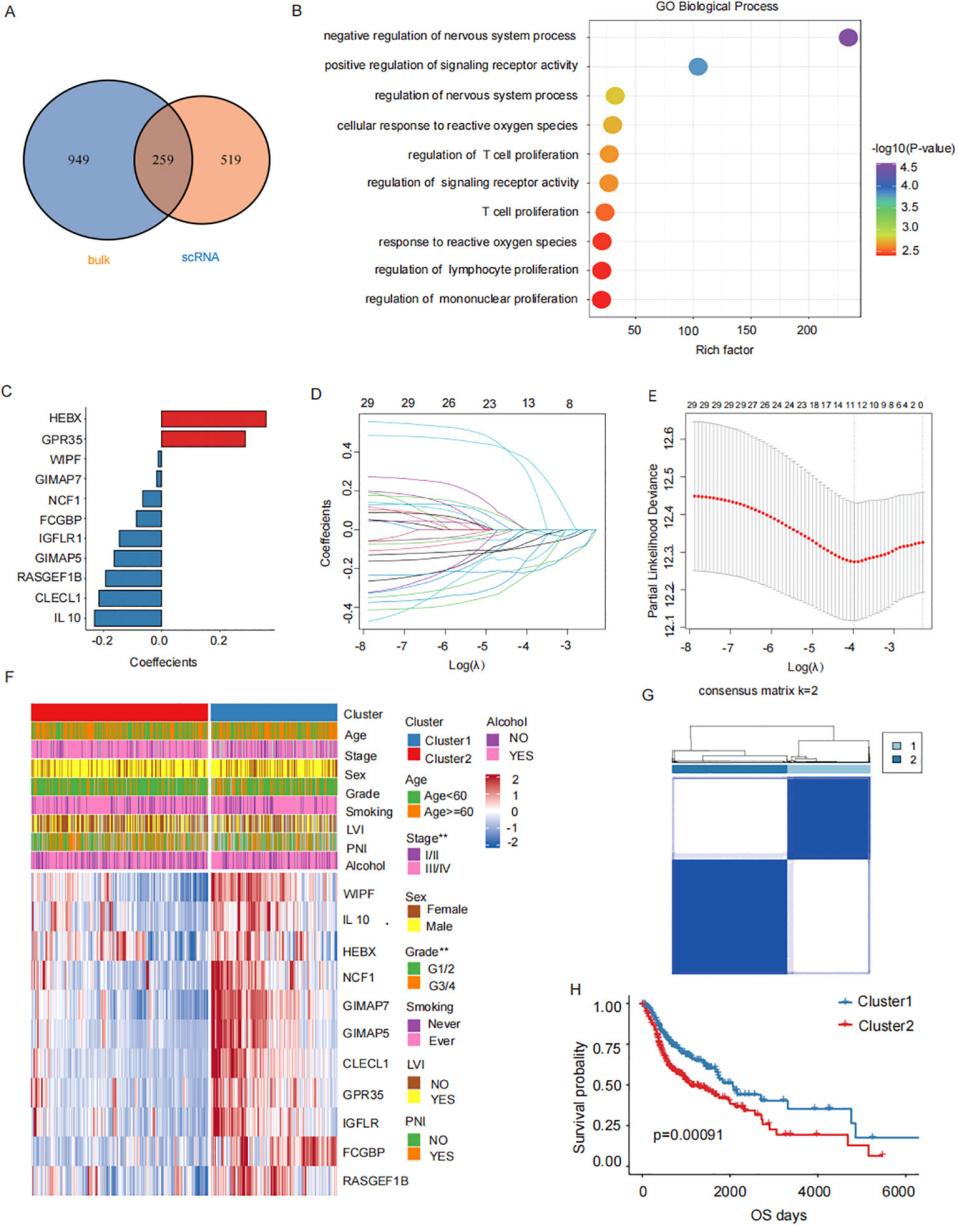
M2 TAMs related prognostic genes. **(A)** Candidate M2 TAMs-related genes(1208 TAMs-specific signature genes from single-cell database GSE150430 and the 778 M2 macrophage-associated genes from bulk database TCGA- HNSCC). **(B)** Functional enrichment analysis of 11 key prognostic factors (T cell proliferation, lymphocyte proliferation). **(C)** Key prognostic factors LASSO regression coefficients. **(D)** LASSO regresses the trajectory of the independent variable, The horizontal axis represents the logarithm of the independent variable Lambda, while the vertical axis represents the coefficients of the independent variables. **(E)** Confidence intervals for each λ value in LASSO regression. **(F)** The upper heatmap depicts the difference in the distribution of clinical characteristics between the two groups. The bottom heatmap shows the distribution of prognostic risk factors expression. **(G)** HNSCC samples were consistently clustered, with 1 and 2 denoting the two subgroups. **(H)** Prognostic survival in two subgroups curves.

The intersection of the 1208 TAMs-specific signature genes from single-cell database and the 778 M2 macrophage-associated genes from bulk database resulted in 259 candidate M2 TAMs-associated genes (**Figure 3A**). Functional enrichment analysis revealed that key prognostic factors were significantly enriched in immune processes such as T cell proliferation and lymphocyte proliferation (**Figure 3B**). Twenty-nine genes associated with clinical prognosis were identified through univariate cox analysis and were furthered narrowed down to 11 key prognostic genes by LASSO Cox regression analysis (**Figures 3C–E**). The HNSCC samples were classified into two subgroups (cluster1 and cluster2), based on the expression of the 11 key factors (**Figures 3F, G**, **Supplementary File 2**). Prognostic survival rates in cluster 2 showed a significantly worse prognosis (**Figures 3H**). The subsequent analysis involved examining the expression levels of these 11 key prognostic genes to establish a prognosis-related signature.

### 3.3 Construction and validation the prognostic gene model of M2 TAMs in HNSCC

The gene coefficients from the linear combination of 11 key prognostic factors were used to define the prognostic signature for each patient, as shown in **Table 2**.

**Table 2:** Key *factor* and corresponding coefficients.

Signature	Coef
FCGBP	-0.087011342
GIMAP5	-0.163148894
WIPF1	-0.011925908
RASGEF1B	-0.192706961
GIMAP7	-0.016669482
IGFLR	-0.145697518
GPR35	0.288017291
NCF1	-0.215696193
CLECL1	-0.215696193
HEXB	0.359694783
IL10	-0.230829119

**Figure 4:**
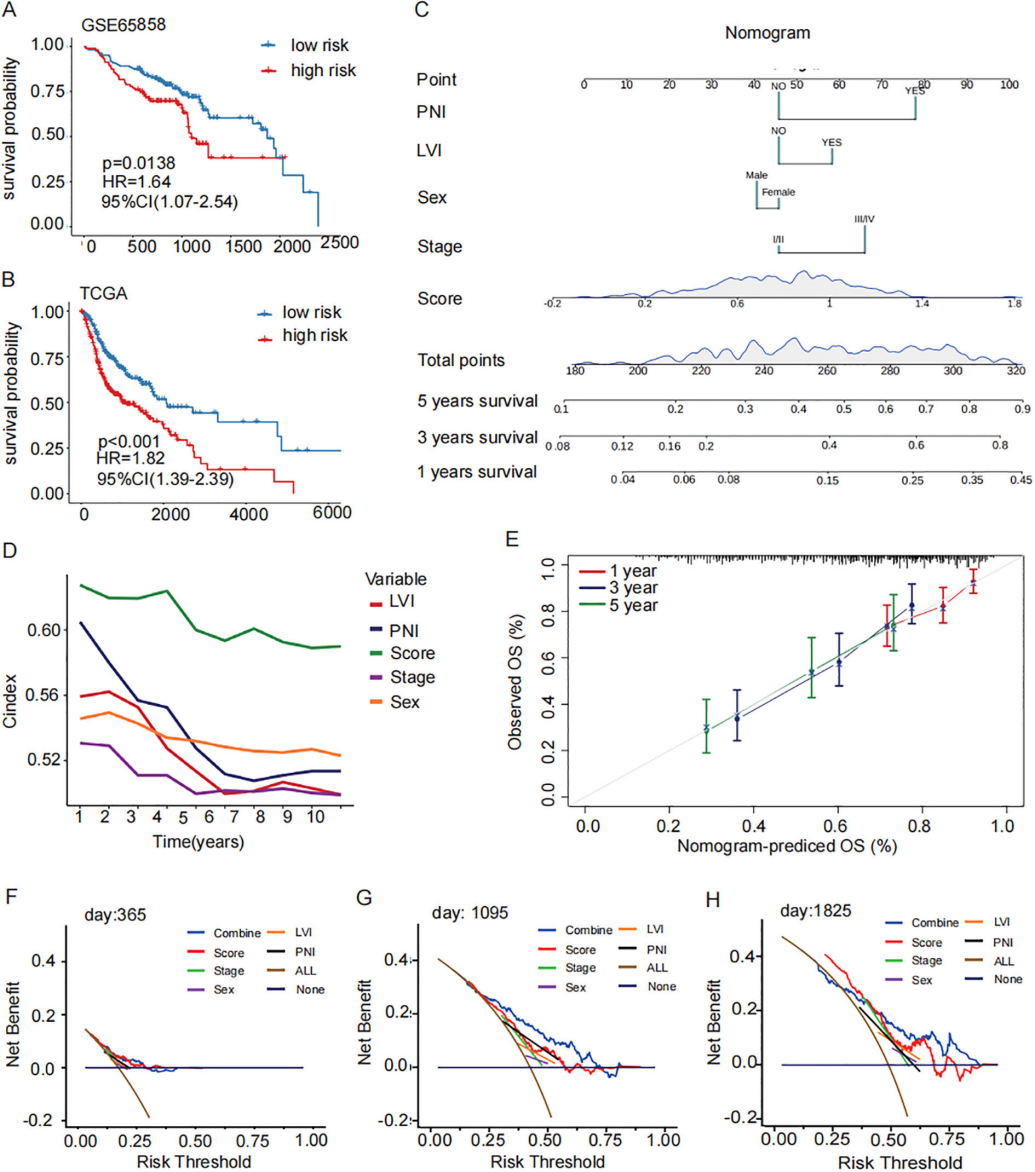
Construction and validation of a prognostic signature for M2 TAMs. **(A, B)** GSE65858 Prognostic survival curves and the prognostic independence analysis for the high- and low-risk groups of the TCGA training set and GSE65858 validation set (log-rank p-value < 0.001). **(C)** nomogram of clinical factors predicts patient survival rate (5 years survival, 3 years survival, 1 year survival). **(D)** C index concordance show the prognostic accuracy of the signature is higher than other clinical pathological indicators. **(E)** Calibration curves for assessing accuracy of the nomogram. The dashed diagonal line in grey represents the ideal model. **(F–H)** calibration curve reveals the demonstrated the reliability of the prognostic signature. OS, overall survival; ROC: receiver operator characteristic.

According to the median value, cases were classified into high-risk group and low-risk group. Kaplan-Meier survival analysis and log-rank tests revealed that patients in the high-risk group had a significantly poorer prognosis in GSE65858 (*P<0.02*, **Figure 4A**). Based on multivariate and univariate Cox regression analysis performed with clinical features and prognosis signature, demonstrated a consistent trend in predicting prognosis. These analyses also confirmed that the prognostic signature was an independent prognostic factor (HR=1.65, *P-value=0.01*, **Supplementary Figures S2B**). The data from GSE65858 further supported the prognosis signature to be an independent prognostic factor (**Figures 2C D**). Patients in the high-risk group had significantly worse overall survival rate in TCGA (*P<0.001*, **Figure 4B**). A nomogram was plotted, incorporating clinical factors such as stage, sex, lymph vascular invasion (LVI), and perineural invasion (PNI) to provide a more comprehensive survival prediction (**Figure 4C**). The calibration curve (**Figures 4F–H**) and decision curve analysis (**Figure 4E**) demonstrated its reliability of the model. The concordance index (c-index) analysis demonstrated that the prognostic signature exhibited higher accuracy than other clinicopathological indicators (**Figure 4D**). Next, the distribution of prognosis signature among clinical pathology characteristics was analyzed.

### 3.4 Association analysis of prognostic signature with clinical and pathologic features

**Figure 5:**
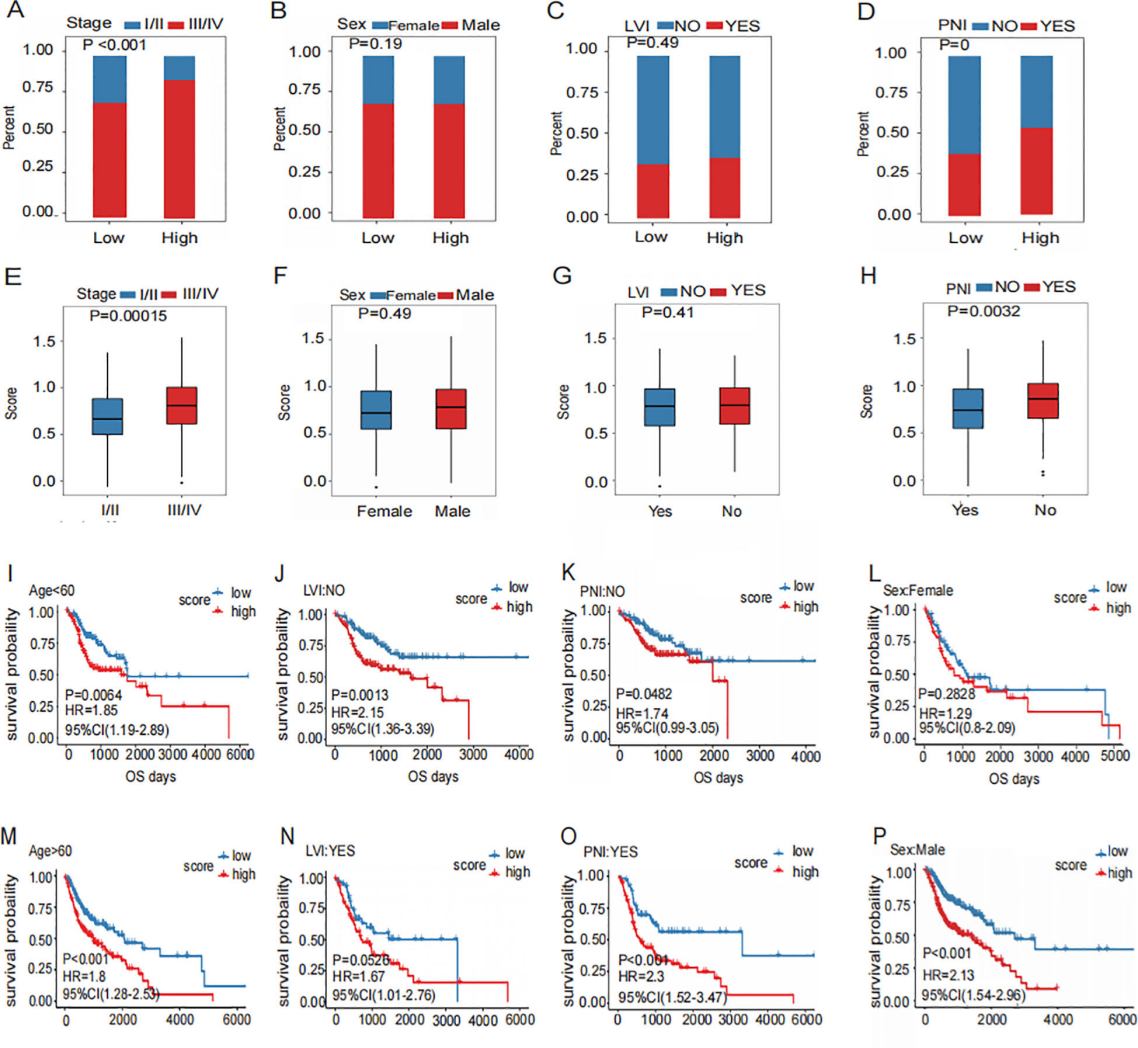
Association of prognostic signature with clinic pathology features.**(A–D)** Distribution of Clinical Characteristics (Stage **(A)**, Sex **(B)**, LVI **(C)** and PNI **(D)**) in High and Low Risk Groups. **(E–H)** Different Distribution of risk scores for subgroups with four clinical characteristics (Stage **(E)**, Sex **(F)**, LVI **(G)** and PNI **(H)**). **(I–P)** Prognostic Survival Curve for High and Low Risk Groups Grouped by four Clinical Characteristics (Stage **(I, M)**, Sex **(J, N)**, LVI **(K, O)** and PNI **(L, P)**).

The distribution of the prognostic signature across clinical pathological characteristics, showed that the proportion of patients with terminal cancer was higher in the high-risk group (**Figure 5A**). However, there was no significant difference between the two groups in terms of gender (**Figure 5B**), or lymphovascular invasion (LVI) (**Figure 5C**). The proportion of patients with perineural invasion (PNI) in the high-risk group was significantly higher in those with advanced stage disease (**Figure 5D**).

The risk scores of patients with PNI were elevated in those with advanced disease (**Figure 5E**). However, no significant difference in risk scores was observed between genders (**Figure 5F**). Furthermore, there was no significant difference in risk scores between patients with or without LVI (**Figure 5G**). Risk scores were significantly higher in patients with PNI (**Figure 5H**). This malignant biological behavior, which appeared frequently in the high-risk group, indicated a poor prognosis.

In different clinical pathological characteristic subgroups, the signatures showed that the prognosis in patients with advanced stage (**Figure 5M**), men (**Figure 5N**), without PNI (**Figure 5K**), without LVI (**Figure 5K**), female (**Figure 5L**), and those with PNI (**Figure 5P**) were poorer. Patients in the high-risk group generally had worse prognosis. On the contrary, patients in the early stage (**Figure 5I**), in women (**Figure 5J**), showed no significant difference in prognosis between high- and low-risk groups. (**Figure 5O**). These findings suggest that the prognostic signature can effectively predict the poor prognosis of HNSCC.

### 3.5 Immune profile in the high-risk and low-risk groups of prognosis signature

**Figure 6:**
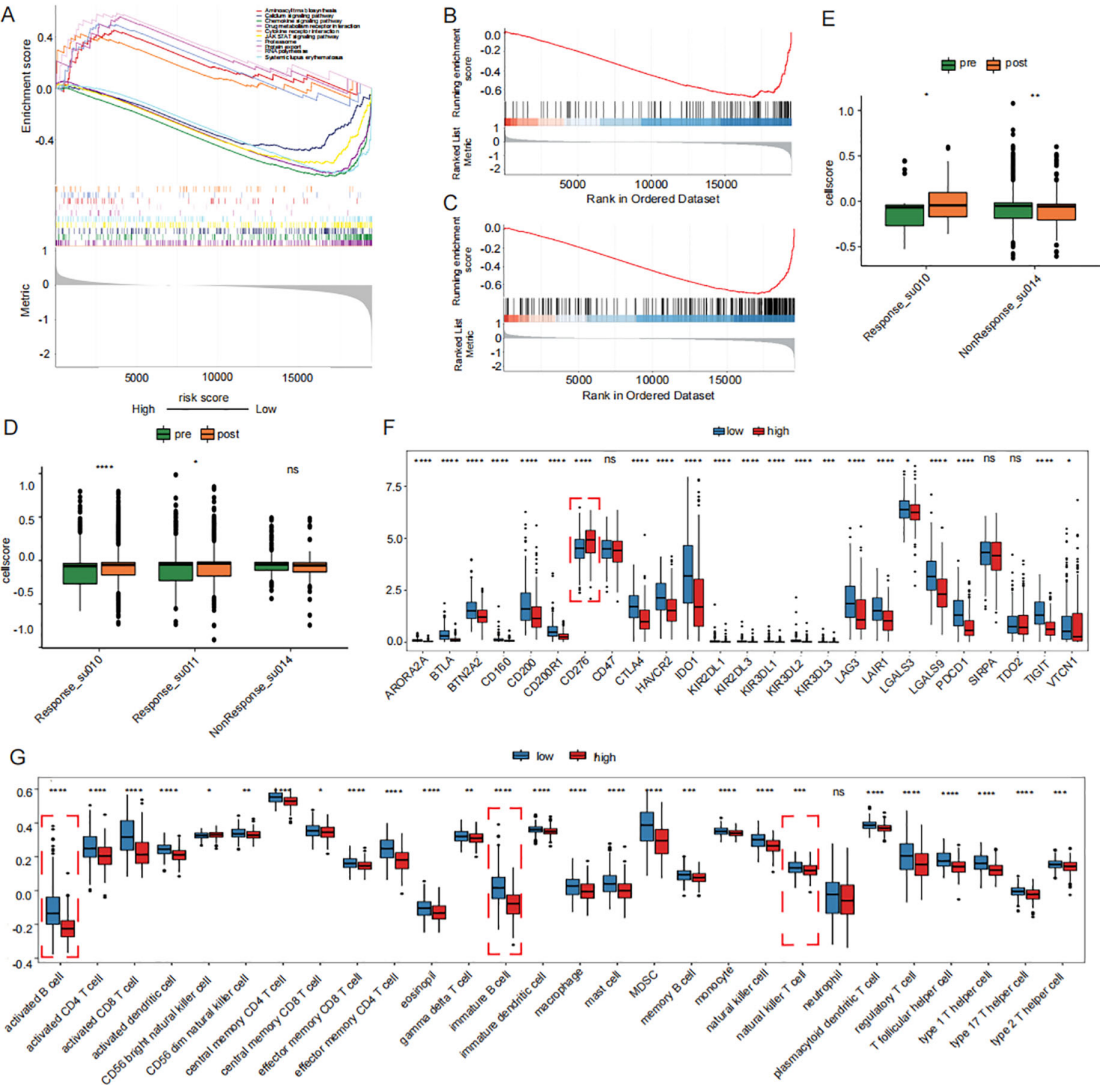
Prognostic signature-associated immunoepidemiogram. **(A)** drug metabolism pathways were significantly activated in the high-risk group in KEGG GSEA analysis. **(B, C)** activation and proliferation of B cells and T cells were markedly enhanced in the low-risk group in GOBP GSEA analysis. **(D)** Distribution of Th17 cell scores before immunotherapy. **(E)** Distribution of CD8 cell scores before immunotherapy. **(F)** Differences in immune checkpoint expression between high and low risk groups. **(G)** Distribution of proportion of immune cell infiltration in high and low risk groups. GSEA: Gene Set Enrichment Analysis.

GSEA analysis revealed that drug metabolism pathways were significantly activated in the high-risk group (**Figure 6A**). Conversely, immune-related biological processes, including the activation and proliferation of B cells and T cells, were markedly enhanced in the low-risk group (**Figures 6B, C**). The immune cell scores were compared before and after treatment. Th17 cells showed a significantly increase in cell scores following immunotherapy in patients who responded to immunotherapy, which can be interpreted as an increase in cellular activity. (**Figures 6D, E**). Activated B cells, immature B cells, and natural killer T cells were significantly more abundant in the low-risk group, whereas the proportion of CD56+ natural killer cells was lower than that in the high-risk group (**Figure 6G**). Subsequently, comparison of immune checkpoint-related gene expression revealed significantly higher expression of CD276 in the high-risk group (**Figure 6F**). The distribution of biomarkers in the high-risk group, as determined by the signature we constructed, can serve as a better indicator for assessing poor prognosis.

### 3.6 Clinical validation of prognosis signature of M2 TAMs

Based on the expression levels of the 11 key prognostic factors and gene coefficients in a linear combination, the prognostic signature of twenty patients was evaluated, as shown in **Table 3**.

**Table 3:** 20 HNSCC Patients’ clinical data(2023-2024).

Patients (n=20)		
gender	n	percent (%)
male	10	50
female	10	50
age		
>60	9	45
<=60	11	55
metastasis		
M0	12	60
M1	8	40
stage		
T1-T2	7	35
T3-T4	13	65
Invasion		
yes	7	35
no	13	65

**Figure 7:**
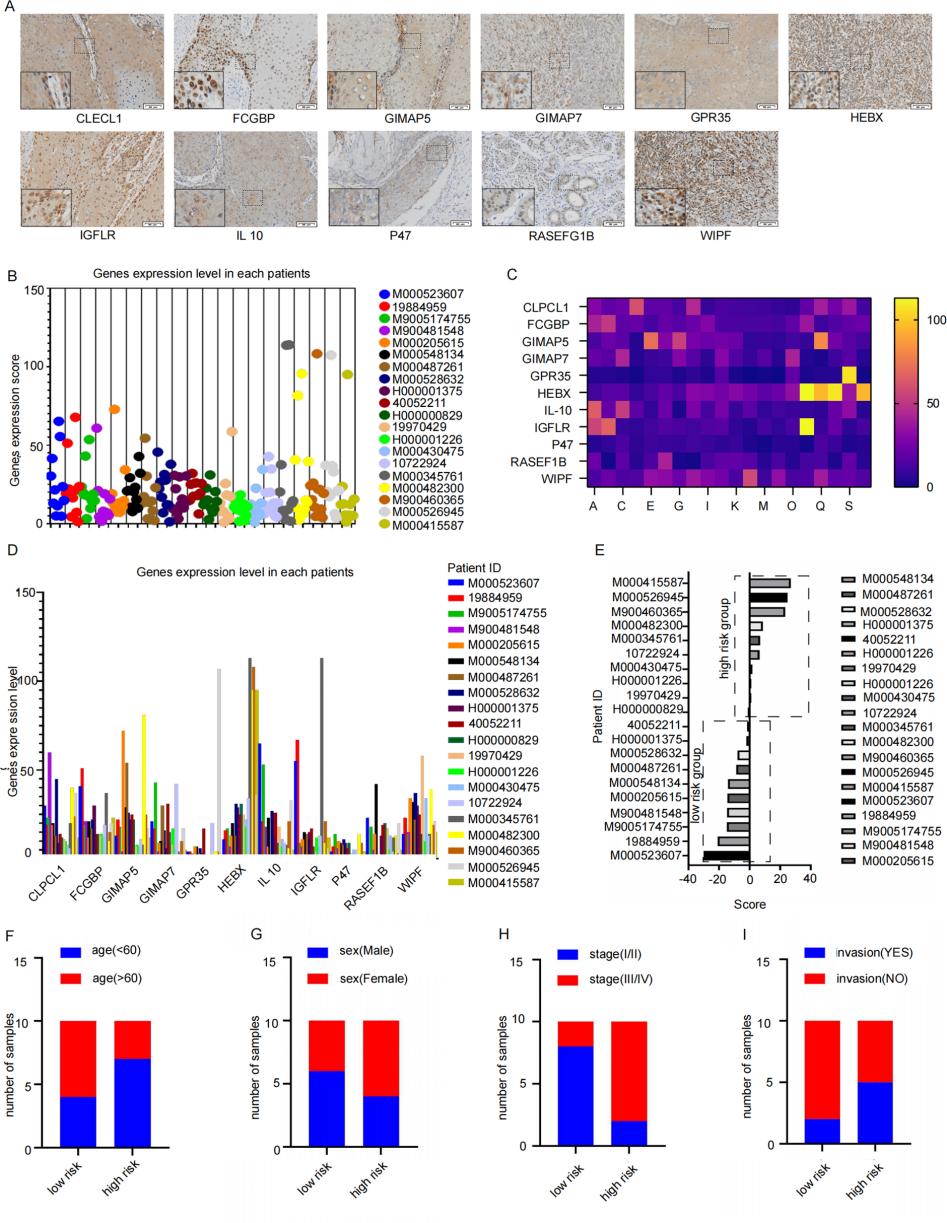
The signature verification of results. **(A)** The Immunohistochemistry quantitative images which were used to analysis by Image J (Twenty patients with HNSCC who underwent surgery between January 2023 and January 2024 provided primary tumor tissues, Scale bar: 50 μm) **(B–D)** the expression level of 11 key factor genes in clinical samples. **(E)** the samples were consistently clustered, with high- and low-risk two subgroups based on the median value of signature. **(F–I)** Distribution of Clinical Characteristics (Stage **(F)**, Sex **(G)**, LVI **(H)** and PNI **(I)**).

Tissue sections from these patients with HNSCC were examined immunohistochemically, and their expression scores were analyzed (**Figure 7A**). The obtained scores were incorporated into constructed signatures to generate predictions (**Table 4**). The gene expression levels were consistent with the key genes predicted by LASSO regression for the prognostic factors (**Figures 7B–D**).

**Table 4:** Signature scores for each sample.

Number	Signature	Number	Signature
1	-35.10636046	11	-4.855251866
2	-25.96694989	12	-3.924183372
3	-19.99509091	13	-3.856082207
4	-19.80199138	14	-3.233356117
5	-19.54928762	15	1.536435724
6	-19.18544045	16	2.065886704
7	-13.88992481	17	3.773816865
8	-12.89721797	18	18.32559862
9	-7.090439303	19	19.99195797
10	-6.704592289	20	21.94864881

According to the constructed prognostic signature, we calculated the risk score for patients in the training set and stratified them into high-risk and low-risk group based on the median risk score (**Figure 7E**). The proportion of patients under 60 years old was higher in the high-risk group and lower in the low-risk group (**Figure 7F**). Furthermore, the high-risk group also had a significantly higher number of stage III/IV patients compared to stage I/II patients (**Figure 7G**). Gender did not show a significantly difference (**Figure 7H**). Additionally, low-risk group had significantly more patients without invasion compared to those with invasion (**Figure 7I**). These findings suggest that the prognosis signature has the potential to predict the prognosis effectively in patients with HNSCC.

## 4 Discussion

Our study revealed that the TAMs are the most abundant subtype among tumor-infiltrating immune cells in HNSCC. Increased TAMs infiltration within the TME has been significantly associated with lymph node metastases and advanced clinical stages in HNSCC. TAMs are broadly polarized into M1 and M2 phenotypes (15). Animal *in vivo* experiments further identified M2 TAMs infiltration as a prognostic indicator for HNSCC progression. In this study, we analyzed the HNSCC specimens and quantified M2 TAMs density. Through single-cell sequencing, we identified M2 macrophage signature genes and subsequently constructed a prognostic risk model based on these genes, which was validated in clinical patient samples.

The identification of robust risk stratification models and prognostic biomarkers is crucial for the accurate prediction of clinical outcomes and the evidence-based optimization of therapeutic interventions (16). We stratified patients with HNSCC into high-risk and low-risk groups according to predefined thresholds for M2 TAMs infiltration density derived from TCGA cohort. Notably, patients with high M2 TAMs infiltration density exhibited significantly poorer overall survival rates compared to those with low infiltration levels (*P<0.001*). Through both multivariate and univariate Cox regression analysis involving clinical features and prognosis signatures, the model demonstrated a consistent trend in predicting prognosis. These findings highlight the critical role of M2 macrophage enrichment as an independent predictor of poor clinical outcomes in HNSCC.

M2-type TAMs are well-documented as a key driver of HNSCC progression (17). Using WGCNA and UMAP analysis, we systematically screened genes from 1208 M2 TAMs across both single-cell and bulk RNA sequencing datasets. The intersection of these genes led to the identification of 11 key prognostic biomarkers. These includeFCGBP (18), GIMAP5 (19), WIPF1 (20), RASGEF1B (21), GIMAP7 (19), IGFLR1 (22), GPR35 (23), NCF1 (24), CLECL1 (25), HEXB (26) and IL10 (27) which may serve as important predictors of HNSCC in tumor microenvironment. FCGBP is likely involved in gel-forming mucins activity (28). Previous studies on GIMAP5 have shown that that its low expression is associated with poor prognosis in lung cancer (19). The gene encoded by Wiskott–Aldrich syndrome protein (WASP) interacting protein family member 1 (WIPF1) participates in actin cytoskeleton organization and polymerization that are associated with cell proliferation and invasion (29). In hepatocellular carcinoma, aberrant expression of circular RNA DHPR promotes tumor growth and metastasis by regulating the RASGEF1B/RAS/MAPK axis (30). Macrophage-related gene expression profiles were curated from the Gene Expression Omnibus (GEO) repository, including GSE65858 and GSE150430 and GSE123813, all of which underwent rigorous quality control.

Single-cell analytical data serve as a critical component for enhancing the robustness of predictive biomarkers. scRNA-seq has emerged as a powerful methodology for dissecting intertumoral heterogeneity by profiling transcriptional landscapes at single-cell resolution. However, it should not be overlooked that the single-cell database GSE65858 was derived from nasopharyngeal carcinoma. Although the above-mentioned tumors are all malignant tumors of squamous epithelial origin and share some core biological characteristics, the biological differences in different disease backgrounds may affect the integration results, which is a limitation of this study.

The prognostic signature was validated as an independent predictor of clinical outcomes in this cohort. Our findings contribute to a deeper understanding of the molecular mechanisms associated with M2 TAMs in HNSCC, uncover the immune profile specific to HNSCC, and offer potential therapeutic targets for intervention in this malignancy. Notably, the immune checkpoint molecule CD276 demonstrated significantly elevated expression in high-risk patients compared to the low-risk cohort (*P<0.05*). These findings suggest CD276 as a potential therapeutic target for immune checkpoint blockade strategies.

In conclusion, this study focused on constructing a novel M2 TAMs-related risk prediction model and identifying 11 risk factors as prognosis indicators of tumor risk in patients with HNSCC. Subsequent efforts should focus on screening core genes and optimizing detection technologies (such as multiplex molecular diagnostic platforms) to promote its translation into a rapid clinical detection tool. By further stratifying the molecular subtypes within the high-risk group, sorted by the prognostic signature, the accuracy of target selection for HNSCC treatment can be improved, providing convenience for new treatments and techniques. We will next focus on further validating the accuracy of the prognostic signature and exploring the relationship between sensitivity to major anticancer drugs and the high-risk prognostic group to enhance its clinical applicability.

## Data Availability

The datasets presented in this study can be found in online repositories. The names of the repository/repositories and accession number(s) can be found in the article/**Supplementary Material**.

## Supplementary material

Supplementary Figure 1 Macrophage survival analysis and M2 macrophage-related genes **(A)** sample clustering tree. **(B, C)** analysis of network topology for various soft-thresholding powers. **(D)** Gene deprograms and module color. **(E)** Module-feature correlation. **(F)** blue model.

Supplementary Figure 2 Construction and validation of M2-type TAM prognostic signature **(A)** DCA decision curves. **(B–E)** GSE65858 Prognostic survival curves and the prognostic independence analysis for the high-risk and low-risk groups of the TCGA training set and GSE65858 validation set.
