# Supplementary figures and images for: Combined single-cell RNA-seq and bulk RNA-seq construction of M2 TAMs signature for predicting HNSCC prognosis and immunotherapy
Source: Front Immunol. 2025 Aug 12;16:1620931. doi: 10.3389/fimmu.2025.1620931 (PMC12378706; doi:10.3389/fimmu.2025.1620931)

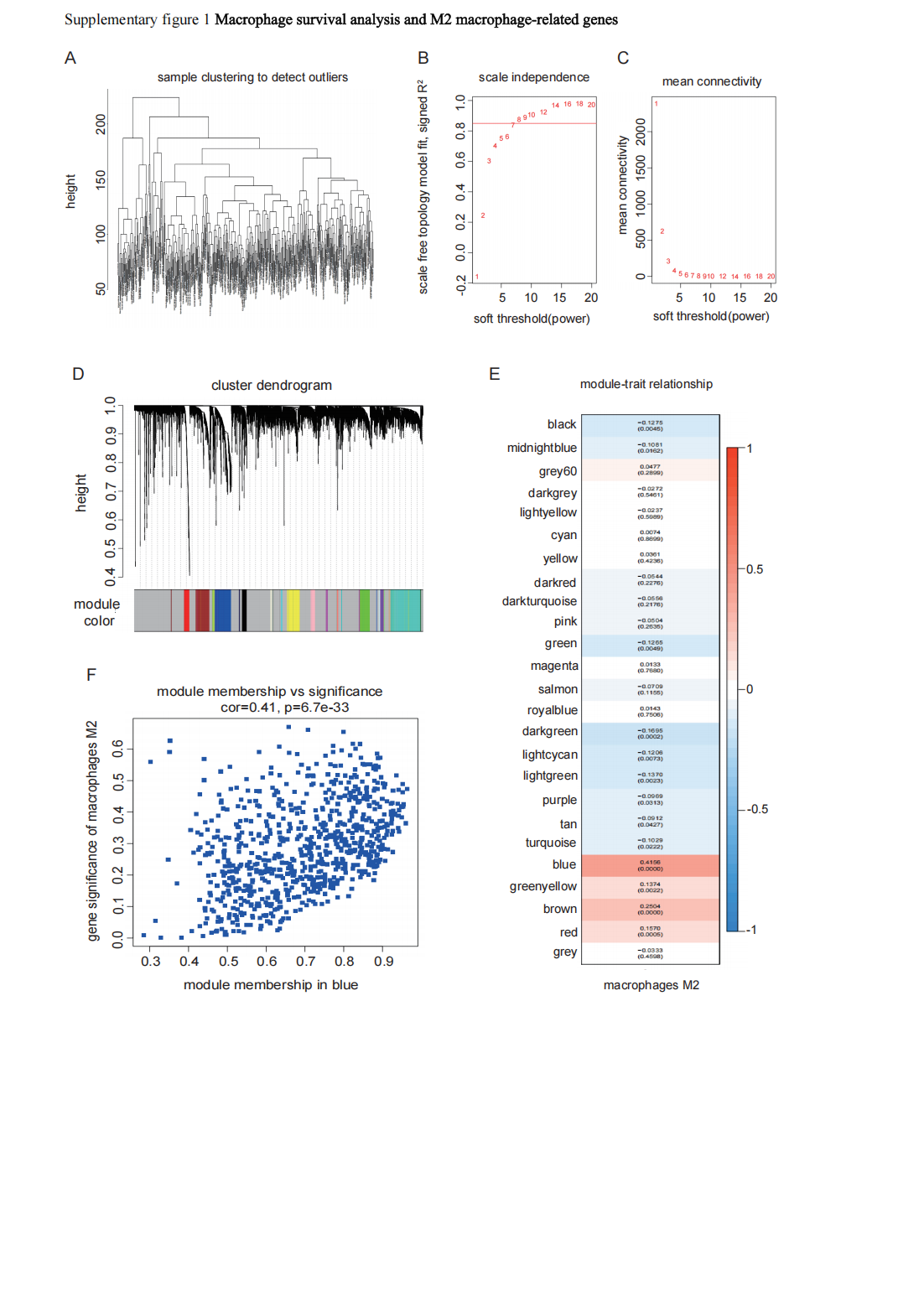

Supplement: Supplementary Figure 1 — Macrophage survival analysis and M2 macrophage-related genes (A) sample clustering tree. (B, C) analysis of network topology for various soft-thresholding powers. (D) Gene deprograms and module color. (E) Module-feature correlation. (F) blue model. [file Image1.tif]

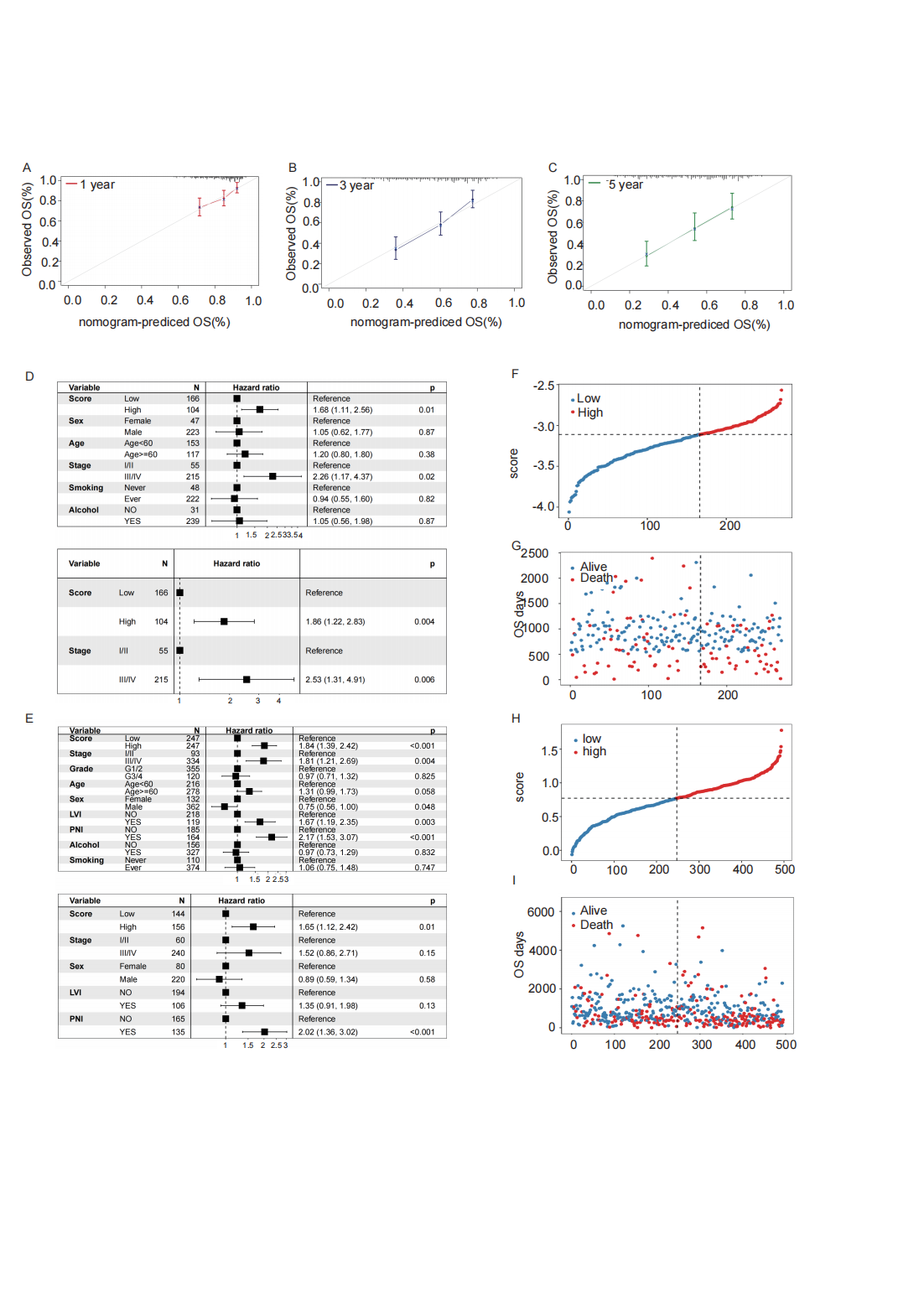

Supplement: Supplementary Figure 2 — Construction and validation of M2-type TAM prognostic signature (A) DCA decision curves. (B–E) GSE65858 Prognostic survival curves and the prognostic independence analysis for the high-risk and low-risk groups of the TCGA training set and GSE65858 validation set. [file Image2.tif]
